# Supplementary material for: Crystal structure and chemical inhibition of essential schistosome host-interactive virulence factor carbonic anhydrase SmCA
Source: Commun Biol. 2019 Sep 5;2:333. doi: 10.1038/s42003-019-0578-0 (PMC6728359; doi:10.1038/s42003-019-0578-0)

Supplementary figures and legends

Supplementary figure 1

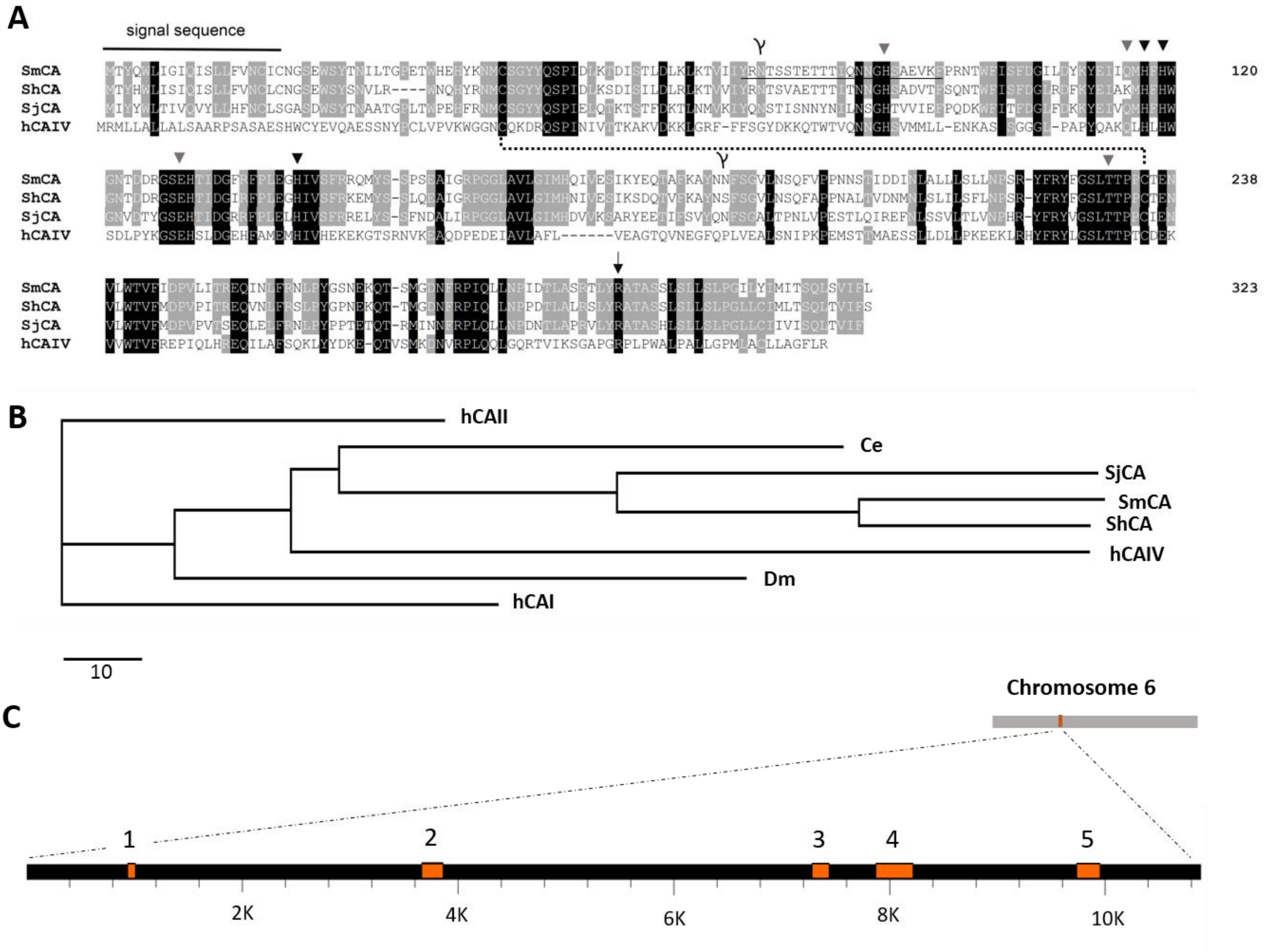

Supplementary figure 1 legend.

SmCA protein and gene organization. A. Alignment of the predicted amino acid sequence of SmCA with other selected CAs using CLUSTAL. Dashes indicate gaps introduced to maximize matching. Identical amino acids across all four sequences are white on a black background and amino acids found in three of four are white on a grey background. A predicted amino terminal 20 amino acid signal sequence in SmCA (^{1}MTYQWLIGIQISLLFVNCIC^{20}) is indicated by the solid line in the upper left. Three conserved zinc binding histidine residues (H^{117}, H^{119}, H^{142}) are indicated by black arrowheads and four highly conserved, critical active site residues (H^{88}, Q^{115}, E^{129}, T^{231}) are indicated by grey arrowheads. A conserved, potential GPI-modification site (R^{295}) is indicated by an arrow.

γ signifies verified glycosylation sites (at residues N^{74} and N^{189}). The dotted line connects C^{45} and C^{235} signifying the presence of a disulfide linkage. Designations (and accession numbers) are as follows: *S. mansoni* CA (SmCA, MK611932); *S. haematobium* ShCA (MK611933); *S. japonicum* SjCA, (CAX74423); Human isoform IV (hCA IV, AAA35625.1). The sequence of the peptide used to generate anti-SmCA antibodies (^{72}YRNTSSTETTTIQNNGHSAEVKF^{94}) is underlined. **B**. Unrooted phylogenetic tree of selected CAs generated by neighbor joining with Accelrys Gene software. The scale bar represents the number of amino acid differences per unit length. Designations (and accession numbers) additional to those given in A, above, are as follows: Ce (*Caenorhabditis elegans*) NP_510674.1, Dm (*Drosophila melanogaster*) NP_523561.1. hCAI (Human isoform 1) NP_001729.1, hCAII (Human isoform 2) AAH14949.1. **C**. Top: Diagrammatic representation of *S. mansoni* chromosome 6 showing the location of the SmCA gene. Bottom: Diagrammatic representation of the SmCA gene. Numbered exons are depicted in orange. Scale numbers at bottom represent kilobases (K).

Supplementary figure 2

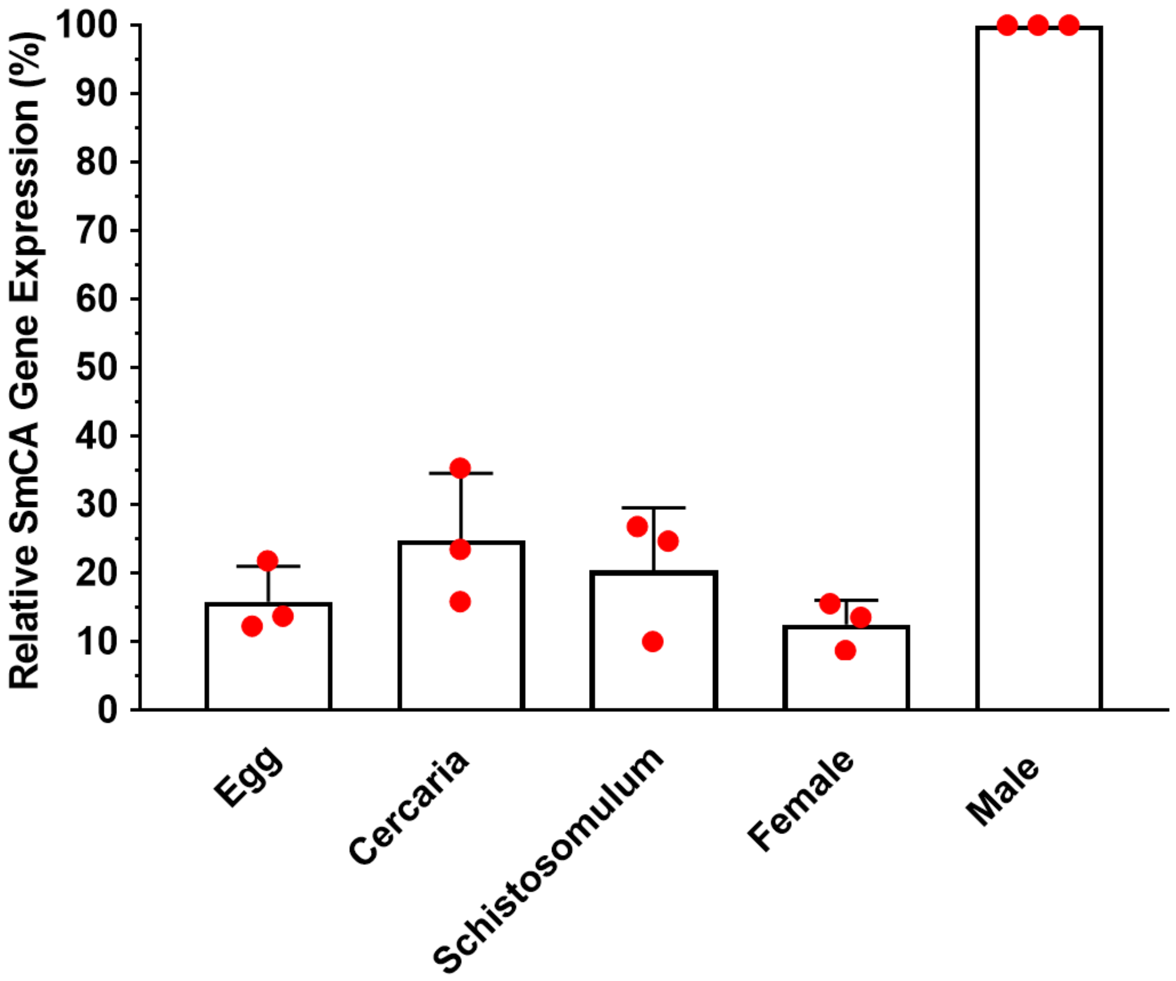

Relative developmental expression of SmCA as measured by RT-qPCR in the parasite life stages listed. Expression in adult males is set at 100%. Red symbols represent source data (i.e. mean values from 3 biological replicates).

Supplementary figure 3

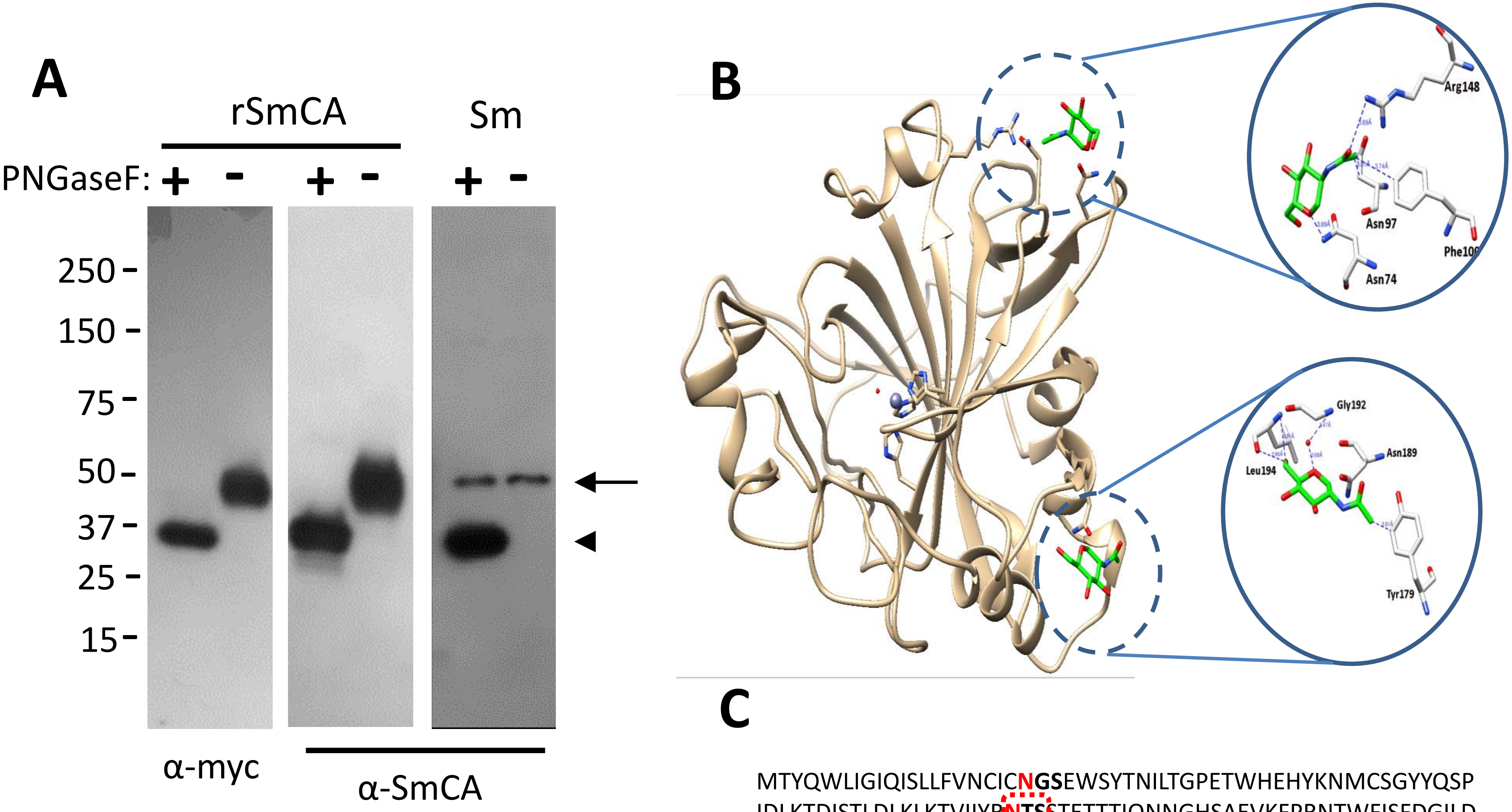

C

MTYQWLIGIQISLLFVNCIC**NGS**EWSYTNILTGPETWHEHYKNMCSGYYQSP
IDLKTDISTLDLKLKTVIIYR**NTS**STETTTIQNNGHSAEVKFPRNTWFISFDGILD
YKYEIIQMHFHWGNTDDRGSEHTIDGFRFPLEGHIVSFRRQMYSSPSEAIGR
PGGLAVLGIMHQIVESIKYEQTAFKAY**NNFS**GVLNSQFVPP**NNST**IDDINLAL
LLSLL**NPS**RYFRYFGSLTTPPCTENVLWTVFIDPVLITREQINLFRNLPYGSNEK
QTSMGDNFRPIQLLNPIDTLASRTLYRATASSLSLLSLPGILYIMITSQLSVIFL

Supplementary figure 3 legend.

Supplementary figure 3. A. Western blot analysis of SmCA glycosylation status. Recombinant SmCA (rSmCA) or adult worm lysate (Sm) was resolved by SDS-PAGE following treatment with PNGase F (+) or after no treatment (-), as indicated. In both cases, untreated protein resolves at ~50kDa (arrow). Following treatment with PNGase F, a new, smaller band (at ~37 kDa) appears (arrowhead). The recombinant protein is detected with α-myc tag antibody (left panel) or anti-SmCA antibody (center panel) and the native protein is detected with anti-SmCA antibody (right panel) **B. Crystal structure determination of the N-glycosylation sites of SmCA**. Ribbon diagram of SmCA structure with circles highlighting sites of N-glycosylation. N-acetyl-D-glucosamine residues (green) are shown covalently bound to Asn^{74} (upper circle) and Asn^{189} (lower circle). Surrounding amino acids are also depicted. **C. Primary sequence of SmCA** with potential N-linked glycosylation sites highlighted. Sites shown by crystal structure analysis to bind carbohydrate (N^{74} and N^{189}) are denoted by red dotted boxes.

Supplementary figure 4

```
hCAII      MSHHWGYGK-HNGPEHWHKDF-PIAKGERQSPVDIDTHTAKYDPSLKPLSVSYD--QATS
4PXX       MSHHWGYGK-HNGPEHWHKDF-PICKGERQSPVDIDTHTAKYDPSLKPLSVSYD--QATS
4HBA       MSHHWGYGK-HNGPEHWHKDF-PICKGERQSPVDIDTHTAKYDPSLKPLSVSYD--QATS
SmCA       NGSEWSYTNILTGPETWHEHYKNMCSGYYQSPIDLKTDISTLDLKLKTVIIYRNTSSTET

hCAII      LRILNNGHAFNVEFDDSQDKAVLKGGPLDGTYRLIQFHFHWGSLDGQGSEHTVDKKKYAA
4PXX       LRILNNGHAFNVEFDDSQDKAVLKGGPLDGTYRLIQFHFHWGSHDGQGSEHTVDKKKYAA
4HBA       LRILNNGHAFNVEFDDSQDKAVLKGGPLDGTYRLIQFHFHWGSLDGQGSEHTVDKKKYAA
SmCA       TTIQNNGHSAEVKFPRNTW-FISFDGILDYKYEIIQMHFHWGNTDDRGSEHTIDGFRFPL

hCAII      ELHLVHWNT-KYGDFGKAVQQPDGLAVLGIFLKVGSAKPGLQKVV-------DVLDSIKT
4PXX       ELHLVHWNT-KYGDFGKAVQQPDGLAVLGIFLKVGSAKPGLQKVV-------DVLDSIKT
4HBA       ELHLVHWNT-KYGDFGKAVQQPDGLAVLGIFLKVGSAKPGLQKVV-------DVLDSIKT
SmCA       EGHIVSFRRQMYSSPSEAIGRPGGLAVLGIMHQIVESIKYEQTAFKAYNNFSGVLNSQFV

hCAII      K--GKSADFTNFDPRGLLPESLDYWTYPGSLTTPPLLECVTWIVLKEPISVSSEQVLKFR
4PXX       K--GKSADFTNFDPRGLLPESLDYWTYPGSLTTPPCLESVTWIVLKEPISVSSEQVSKFR
4HBA       K--GKSADFTNFDPRGLLPESLDYWTYPGSLTTPPCLESVTWIVLKEPISVSSEQVLKFR
SmCA       PPNNSTIDDINLALLLSLLNPSRYFRYLGSLTTPPCTENVLWTVFIDPVLITREQINLFR

hCAII      KLNFNGEGEPEELMVDNWRPAQPLKNRQIKASFK------
4PXX       KLNFNGEGEPEEPMVDNWRPAQPLKNRQIKASFK------
4HBA       KLNFNGEGEPEELMVDNWRPAQPLKNRQIKASFK------
SmCA       NLPYGS-NEKQTSMGDNFRPIQLLNPIDTLASRTLYRATA
```

SmCA sequence homology and structural comparison. Alignment of residues 21 through 298 of SmCA with that of full length hCA II and two hCA II mutants (4PXX and 4HPA) using CLUSTAL. Differences between hCA II and the mutants are highlighted in red.

Figure 3B-Full image

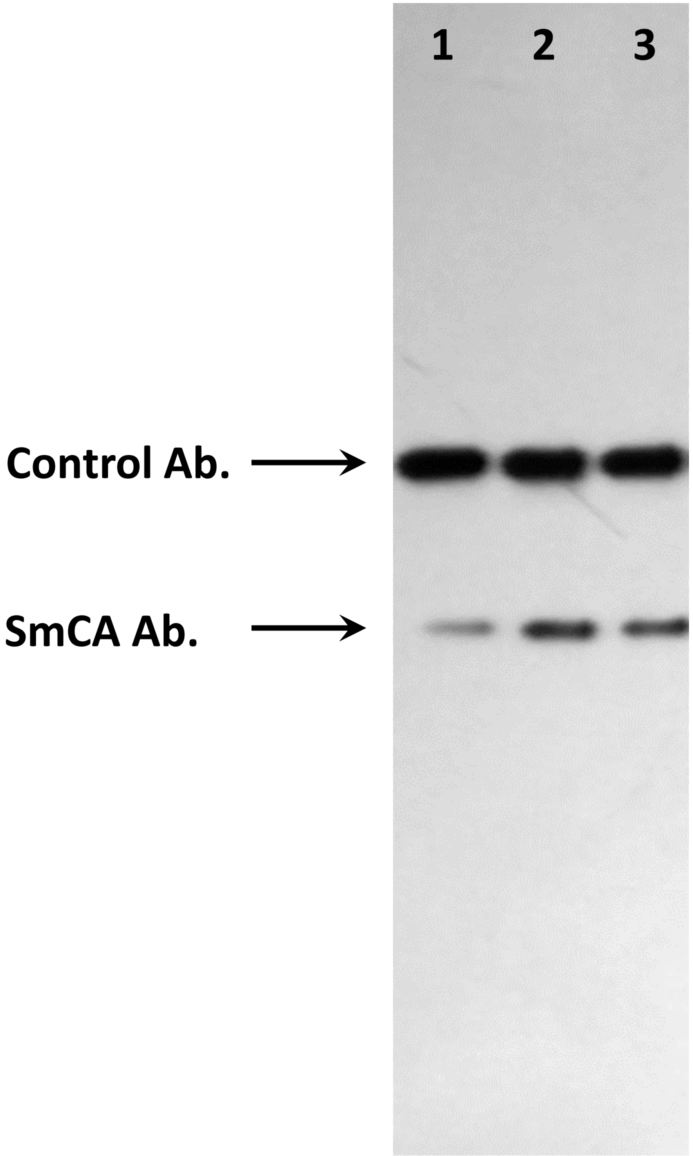

Parasite extracts following:

1. SmCA siRNA treatment.
2. Control siRNA treatment.
3. No treatment.

Figure 5B-Full image

Western blot showing rSmCA (lanes a) and native SmCA (lanes b) on the same gel

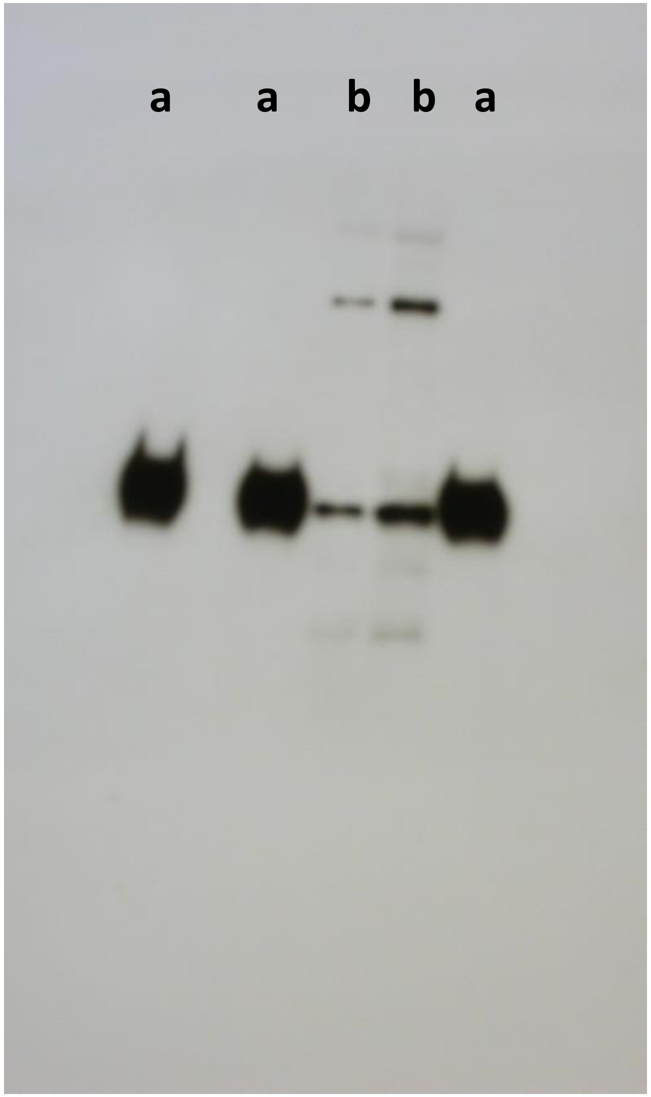

Supplement: Supplementary file 1 — Supplementary Information [file 42003_2019_578_MOESM1_ESM.pdf]
